# Opportunities and limits of combining microbiome and genome data for complex trait prediction

**DOI:** 10.1186/s12711-021-00658-7

**Published:** 2021-08-06

**Authors:** Miguel Pérez-Enciso, Laura M. Zingaretti, Yuliaxis Ramayo-Caldas, Gustavo de los Campos

**Affiliations:** 1 ICREA, Passeig de Lluís Companys 23, 08010 Barcelona, Spain; 2 Centre for Research in Agricultural Genomics (CRAG), CSIC-IRTA-UAB-UB, 08193 Bellaterra, Barcelona Spain; 3 Animal Breeding and Genetics Program, Institute for Research and Technology in Food and Agriculture (IRTA), Torre Marimon, 08140 Caldes de Montbui, Barcelona Spain; 4 Dept. of Epidemiology & Biostatistics, and Dept. of Statistics & Probability, Michigan State University, East Lansing, MI 48824 USA

## Abstract

**Background:**

Analysis and prediction of complex traits using microbiome data combined with host genomic information is a topic of utmost interest. However, numerous questions remain to be answered: how useful can the microbiome be for complex trait prediction? Are estimates of microbiability reliable? Can the underlying biological links between the host’s genome, microbiome, and phenome be recovered?

**Methods:**

Here, we address these issues by (i) developing a novel simulation strategy that uses real microbiome and genotype data as inputs, and (ii) using variance-component approaches (Bayesian Reproducing Kernel Hilbert Space (RKHS) and Bayesian variable selection methods (Bayes C)) to quantify the proportion of phenotypic variance explained by the genome and the microbiome. The proposed simulation approach can mimic genetic links between the microbiome and genotype data by a permutation procedure that retains the distributional properties of the data.

**Results:**

Using real genotype and rumen microbiota abundances from dairy cattle, simulation results suggest that microbiome data can significantly improve the accuracy of phenotype predictions, regardless of whether some microbiota abundances are under direct genetic control by the host or not. This improvement depends logically on the microbiome being stable over time. Overall, random-effects linear methods appear robust for variance components estimation, in spite of the typically highly leptokurtic distribution of microbiota abundances. The predictive performance of Bayes C was higher but more sensitive to the number of causative effects than RKHS. Accuracy with Bayes C depended, in part, on the number of microorganisms’ taxa that influence the phenotype.

**Conclusions:**

While we conclude that, overall, genome-microbiome-links can be characterized using variance component estimates, we are less optimistic about the possibility of identifying the causative host genetic effects that affect microbiota abundances, which would require much larger sample sizes than are typically available for genome-microbiome-phenome studies. The R code to replicate the analyses is in https://github.com/miguelperezenciso/simubiome.

**Supplementary Information:**

The online version contains supplementary material available at 10.1186/s12711-021-00658-7.

## Background

The relevance of microbial ecosystems associated with humans and animals for health and production is now widely recognized, e.g., [1–6]. The fraction of phenotypic variance for a given trait that is explained by the microbiome has been estimated to quantify its influence and has been named ‘microbiability’ ($b^{2}$) [7], in symmetry with the classical ‘heritability’ ($h^{2}$) concept [8]. Previously, the term "hologenome" was coined to describe the joint action of the genome and the microbiome on a phenotype [9].

Numerous microbes are responsible for diseases, e.g. sepsis in humans, and they have been used for diagnoses for many years [10]. Yet, a consequence of the microbiability being larger than zero is that the whole microbiome can be used to predict complex phenotypes, regardless of whether it is a disease or a production trait. This is an important issue since the use of microbiome data has the potential to alter how medical diagnoses in humans or management and breeding decisions in agricultural species are performed.

Several studies have demonstrated the potential value of microbiome data for the prediction of complex-traits. For example, Rothschild et al. [11] showed that microbiome data can be used to improve accuracy in the prediction of obesity and many other phenotypes in humans. Likewise, Lloyd-Price et al. [12] showed that microbiome data can predict future outbursts of bowel disease in humans. Various studies have shown the power of microbiome data to predict methane emission and feed efficiency in cattle [4, 13–15], feed efficiency and carcass traits in pigs [16, 17] and in poultry [18]. In addition, microbiota data from the rhizosphere has been used to predict various plant phenotypes (e.g., crop yield and diseases) [19]. Simultaneously, since the groundbreaking study of Meuwissen et al. [20], prediction of complex traits using genomic information has been embraced in both plant [21] and animal breeding [22], as well as in human genetics [23]. Therefore, combining the host’s genome and microbiome information is a natural next step to improve the prediction of complex traits, a topic that is currently receiving much attention [16, 24].

It is also important to realize that the composition of the microbiome can be affected by the host’s genome. Wang et al. [25] argued that it is evolutionarily justified for the microbiome to be under partial host genetic control since a non-negligible fraction of the cells in an adult body is made up of microbes, especially in the gut. Beginning with the seminal work by Pomp’s team [26], several studies have confirmed the relationship between the host’s genotype and microbiome composition, e.g., [25, 27, 28]. These microbiome genome-wide association studies (MWAS) suggest that microbiome abundances can be treated as any other complex trait in humans or livestock [27]. For example, Crespo-Piazuelo et al. [29] and Ramayo-Caldas et al. [30, 31] identified several quantitative trait loci (QTL) that modulate bacterial and eukaryotic communities in the gut of pigs and in rumen. Although the ‘heritability’ of individual amplicon sequence variants (ASV) or operational taxonomic units (OTU) is typically low, considering the whole microbiome simultaneously should increase power of MWAS [32]. In addition, although microbiome heritabilities vary according to the taxa level considered, they usually increase as we move up from quasi-species to genus or family levels, e.g., [33]

Large-scale studies in humans suggest a predominant role of the environment in shaping the gut microbiome [11]. However, regardless of the relative importance of genetic and environmental factors in shaping the microbiota, microbiome composition per se can have a predictive value. Yet, the use of microbiota for the prediction of future phenotypes or disease outcomes requires some level of stability of the microbiome over time. In the case of the gastrointestinal tract, microbiota colonization starts at birth, when vertical transmission occurs through the mother’s birth canal. Then, microbiota diversity and richness tend to increase as the host ages, to stabilize at adulthood [34, 35]. In ruminants, the microbial populations that inhabit the rumen appear progressively after birth and partially persist throughout life [36].

As noted, the genome-microbiome-phenome is a complex system but quantifying the relationships between host-genome, microbiota, and phenotypes is important for the effective use of microbiome data for prediction of complex traits. Overall, although there are many published reports, we still lack detailed guidelines on the joint use of microbiome and genome information for the prediction of complex traits and on the reliability of parameter inferences. The number of genes that affect microorganism abundance and that can be confidently identified, and the number of microorganism taxa that can influence a given phenotype remain unknown. With this work, our aim was to contribute to this important topic by focusing on three inter-related questions:

1. How useful can the microbiome be for prediction of complex traits?
2. Are microbiability estimates reliable?
3. Can the underlying biological genome-microbiome-links be inferred at a system’s level? On a more refined level, the question that we aimed to address is whether microbiome groups (e.g., OTU or genera) with sizable causal effects on phenotypes can be identified with the typical size of current microbiome data sets?

We address these questions via a novel simulation strategy that uses real microbiome and genotype data as inputs and by proposing a variance-component approach that, in the spirit of mediation analyses, quantifies the proportion of phenotypic variance explained by the genome and the microbiome. Importantly, the approach allows simulation of a partial control of the host’s genome on the microbiome. This is accomplished using a partial permutation approach that preserves the distribution of the genome and the microbiome. For the analyses, we used Bayesian Reproducing Kernel Hilbert Space (RKHS [37]) and Bayes C [20] approaches. RKHS is similar to genomic best linear unbiased prediction (GBLUP) [38], while Bayes C is a variable selection approach that can account for the possibility that some or all the features available in the genome or the microbiome have no effect on the trait of interest. We investigated the three above-mentioned questions across diverse causal scenarios that examined the links between host genomes and microbiomes, and their relations with phenotype for a complex trait.

## Methods

### Causative scenarios considered

**Fig. 1:**
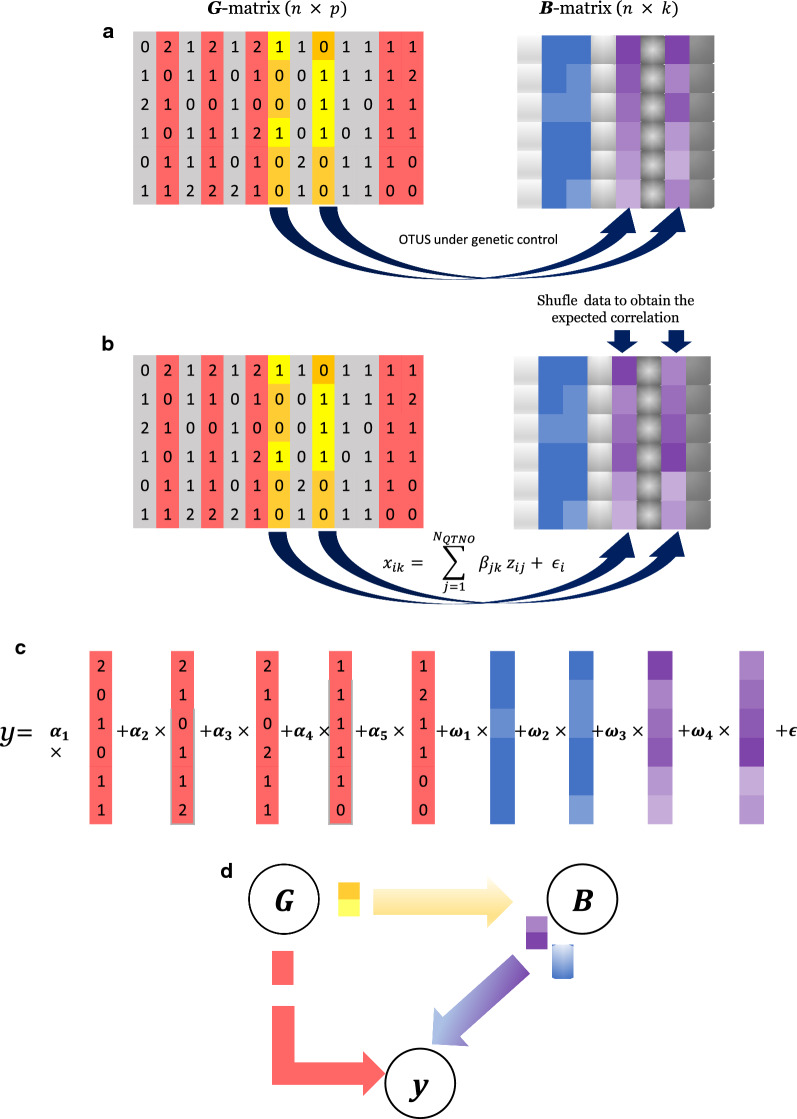
Representation of the scenarios evaluated. G: genome, typically comprises SNP genotype data; B: microbiome; y: phenotype of interest; arrows indicate causality. An arrow from G to y indicates that there is a subset of elements of G (causative SNPs) that influence y; an arrow from G to B indicates that there is a subset of G that influences a subset of abundances in B, which, in turn, may also influence y. An arrow departing from B indicates there is a subset of microbial abundances (the causative abundances) that influence y. The SNPs that affect B need not necessarily be the same as SNPs that affect y directly in the Recursive scenario. Note that B can contain one or more sets of abundances such as archaea and bacteria communities, or different time or site sampling points. Without loss in generality, we assume B is a single community

Because the exact nature of the links between the genome (G), microbiome (B), and phenotype (y) is largely unknown and will likely vary from case to case, we used the six generic causal models (‘scenarios’) illustrated in Fig. 1 to shed light on the nature of the genome-microbiome-phenome links. In the ‘Null’ scenario, there is no link between any of the data-layers; while this scenario is unlikely, it serves as an ‘overall null hypothesis’ and it is useful to assess potential biases in parameter estimates. The ‘Genome’ scenario assumes that only the genome affects the phenotype. In turn, only the microbiome has a direct effect on the phenotype in the ‘Microbiome’ and ‘Indirect’ scenarios. In contrast to the ‘Microbiome’ scenario, the ‘Indirect’ scenario allows for some of the causative abundances to be controlled genetically, which is similar to a scenario in which a phenotype is directly controlled by gene expression levels, and where gene expression is in turn genetically controlled [39, 40]. The ‘Joint’ scenario is the simplest configuration for a trait that is under the influence of both the genome and the microbiome. It assumes that the microbiome and the genome are independent and that their effects on the phenotype are also independent. This is the most widely assumed scenario, implicitly, or explicitly, in the literature, e.g., [4, 11, 16]. The ‘Recursive’ scenario is similar to the ‘Joint’ scenario but it accounts for the possibility that some causative OTU may be under partial genetic control by the host. Therefore, in this scenario, the genome has both direct and indirect (microbiome-mediated) effects on the phenotypes. It should be noted that the ‘Recursive’ model does not assume that the same SNPs have both direct and indirect effects, or that all OTU abundances are under genetic control.

The causal models depicted in Fig. 1 were used to simulate genome-microbiome-phenotype data under different configurations regarding the number of causative loci (QTN) and the number of OTU with effects on the phenotypes, as well as the number of OTU that are affected by the host’s genome. Tables 1 and 2 summarize the simulation models and parameter values chosen.

**Table 1:** Definition of the evaluated scenarios and of the chosen parameters

Scenario	Abbreviation	${\varvec{N}}_{{{\varvec{QTN}}}}$	${\varvec{N}}_{{{\varvec{OTU}}}}$	${\varvec{N}}_{{{\varvec{OTU}}\left( {\varvec{g}} \right)}}$	$h^{2}$	$b^{2}$
Null	0	–	–	–	0	0
Genome	G	100	0	0	$r^{2}$	0
	G500	500	0	0	$r^{2}$	0
Microbiome	M	0	25	0	0	$r^{2}$
Indirect	I	0	25	25	0	$r^{2}$
Joint	J	100	25	0	$r^{2}$*/2*	$r^{2}$*/2*
	J500	500	25	0	$r^{2}$*/2*	$r^{2}$*/2*
Recursive	R	100	25	25	$r^{2}$*/2*	$r^{2}$*/2*
	R500	500	25	25	$r^{2}$*/2*	$r^{2}$*/2*

$N_{QTN}$: number of SNPs with a direct causal effect on the phenotype y; $N_{OTU}$: number of OTU with a direct effect on y; $N_{OTU\left( g \right)}$: number of OTU with a direct effect on y that are genetically determined, i.e., they are a subset of $N_{OTU}$; $h^{2}$ is heritability, $b^{2}$ is microbiability, and $r^{2} = h^{2} + b^{2}$. For $r^{2}$, values of 0.50 and 0.25 were considered. Causative OTU and SNPs were randomly sampled

**Table 2:** Scenarios used to evaluate sensitivity of predictive accuracy to the number of causative OTU

Scenario	Abbreviation	${\varvec{N}}_{{{\varvec{QTN}}}}$	${\varvec{N}}_{{{\varvec{OTU}}}}$	${\varvec{N}}_{{{\varvec{OTU}}\left( {\varvec{g}} \right)}}$	$r^{2}$	$h^{2}$	$b^{2}$
Joint	J10	100	10	0	0.50	0.25	0.25
	J100	100	100	0	0.50	0.25	0.25
	J250	100	250	0	0.50	0.25	0.25
Recursive	R10	100	10	5	0.50	0.25	0.25
	R100	100	100	50	0.50	0.25	0.25
	R250	100	250	125	0.50	0.25	0.25

Symbols are the same as in Table 1

Causative OTU and SNPs were randomly sampled

### A novel data-driven strategy to simulate microbiome-genome-phenotype experiments

Ample literature and software are available on the simulation of ‘standard’ complex phenotypes, e.g., [41–44]. However, these algorithms are not suited for some of the scenarios presented in Fig. 1. Two issues make the simulation of the scenarios shown in Fig. 1 challenging: (i) microbiome data follow zero-inflated highly leptokurtic multivariate distributions [45, 46] and it is not obvious how to sample from these distributions *conditional* on genome data, as is required in the ‘Recursive’ and ‘Indirect’ scenarios; and (ii) in the absence of large-scale published—and public—datasets, it is difficult to obtain accurate estimates of key parameters, such as microbiability, to use in the simulations. To circumvent, or at least to alleviate, these constraints, we used publicly available real data [4, 13] for both G and B.

**Fig. 2:**
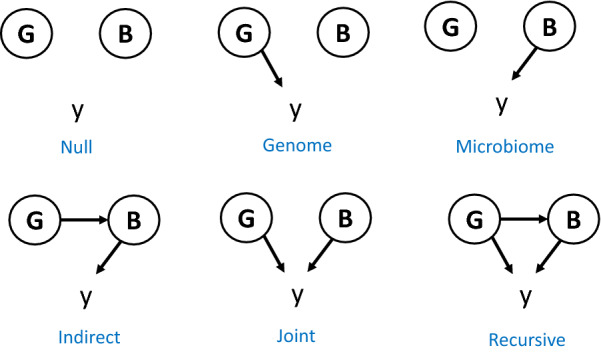
Simulation scheme for the Recursive scenario (Fig. 1). **a** Real input data comprises p genotypes (${\mathbf{G}}$ matrix) and k taxa abundances (${\mathbf{B}}$ matrix). SNPs in grey are neutral, those in red act directly on the phenotype y, and those in yellow/orange influence some OTU abundances (marked in magenta color in ${\mathbf{B}}$ matrix); abundances in blue are not genetically controlled. **b** Given simulated effects, a genotypic value for abundance is obtained via Eq. (2). To obtain the required heritability, abundances in magenta are reordered; to simplify visualization, high abundances (represented by a darker color) are associated with genotype ‘1’. A single SNP is shown as causative for both OTU but there is no limit in practice. **c** The phenotype is simulated by adding the genome and the microbiome contributions plus a residual. **d** The general causal diagram

Simulation under the ‘Joint’ scenario is straightforward since it assumes that G and B act independently (see below). Simulation under the ‘Recursive’ and ‘Indirect’ scenarios is not that obvious because causative abundances are under genetic control and a link must exist between G and B. We solved this by rearranging abundances within individuals such that the desired correlation between abundance and individual’s genotypes was attained (see the Algorithm in Box 1 and the R-code at https://github.com/miguelperezenciso/Simubiome/blob/master/sortCor.R). This strategy has the important advantage that the distribution of abundances is not changed compared to the observed one. Figure 2 recapitulates the simulation strategy. The R code to replicate the analyses is available at https://github.com/miguelperezenciso/simubiome).

### Simulation details

We simulated the joint influence of the genome and the microbiome on a quantitative trait by adding their contributions plus random noise:

1 $$y_{i} = \mathop \sum \limits_{j = 1}^{{N_{QTN} }} \alpha_{j} z_{ij} + \mathop \sum \limits_{k = 1}^{{N_{OTU} }} \omega_{k} x_{ik} + \varepsilon_{i} ,$$

where $y_{i}$ is the $i$-th individual record, $\alpha_{j}$ is the genetic effect of $j$-th causal SNP (QTN), with $j$ = 1 to $N_{QTN}$, which is the number of QTN; $z_{ij}$ is the genotype of the $i$-th individual for the $j$-th SNP coded as -1, 0 and 1 (strict additivity was assumed for all QTN); $\omega_{k}$ is the linear effect of the log-transformed abundance of the $k$-th OTU ($x_{ik}$), with $k$ = 1 to $N_{OTU}$, which is the number of abundances that influence the phenotype; and $\varepsilon_{i}$ is a normally distributed residual. The effect of an OTU can be interpreted as the expected change in phenotype per unit increase in the log-transformed abundance of the OTU. Since abundances are on the log scale, this is equivalent to multiplicative effects of abundance on phenotype. Equation (1) is valid for all scenarios in Fig. 1, except that the term involving markers $\mathop \sum \nolimits_{j = 1}^{{N_{QTN} }} \alpha_{j} z_{ij}$ is removed in the ‘Microbiome’ and ‘Indirect’ scenarios, while the term $\mathop \sum \nolimits_{k = 1}^{{N_{OTU} }} \omega_{k} x_{ik}$ is removed in the ‘Genome’ scenario.

For the ‘Indirect’ and ‘Recursive’ scenarios, variation in abundances ($x$) that is explained by the genome must also be modeled (Fig. 1). Again, we can resort to a linear model in which the log-transformed abundance is treated as a standard complex phenotype:

2 $$x_{ik} = \mathop \sum \limits_{j = 1}^{{N_{QTN\left( k \right)} }} \beta_{jk} z_{ij} + \epsilon_{i} ,$$

where $x_{ik}$ is the log-transformed abundance level of the $k$-th OTU that is under partial genetic control for the $i$-th individual, $\beta_{jk}$ is the genetic effect of the $j$-th QTN on $k$-th abundance, and $z_{ij}$ is the genotype of the $i$-th individual for the $j$-th SNP. The sum is across the QTN that affect abundance of the $k$-th OTU, $j$ = 1 to $N_{QTN\left( k \right)}$. Note that abundances $x_{ik}$ in Eq. (2) are a subset of those in Eq. (1). Other non-causative abundances may also be under genetic control but that is irrelevant for our purpose. Based on this model, phenotype under the ‘Recursive’ scenario was simulated via a two-step procedure by first simulating abundances ($x$) using Eq. (2), followed by simulating phenotype using Eq. (1) given the abundances obtained.

We used real genome and microbiome data as input for the simulation procedure. We downloaded the abundance of 4018 OTU from dairy cattle rumen (N = 750 [4]). A pseudo-count equal to one was added to zero abundances and all abundances were then total-sum scaled and log-transformed, which resulted in much less leptokurtic and asymmetric distributions than original raw abundances. In Eqs. (1) and (2), $x_{ik}$ represents log-transformed abundances.

High-density array genotypes for 750 Holstein cows were downloaded from [13]. To prune SNPs and facilitate computation, 35% of all SNPs (i.e. 32,204) were retained based on a minimum allele frequency of 0.01 and a maximum missing genotypes percentage of 1%. The few missing values were simply imputed with the mean.

Causative OTU and SNPs were randomly sampled, irrespective of their frequency. Thirty simulation replicates per scenario were simulated. Under the ‘Joint’ scenario, which assumes independence between G and B, we can simply sample the list of causative SNPs and abundances, simulate their effects, and apply Eq. 1 to generate phenotype values given the observed genotypes and abundances. In the case of ‘Recursive’ and ‘Indirect’ scenarios, it is not so obvious because we need to sample abundances that are under genetic control and a link must exist between G and B (Eq. 2). We solved this issue by rearranging abundances of a given OTU between individuals such that the desired correlation between abundance and individual’s genotypes is attained. This strategy has the important advantage that the distribution of abundances is not changed. Suppose ${{\varvec{\upgamma}}}_{ik} = \mathop \sum \nolimits_{j = 1}^{{N_{QTNO} }} \beta_{jk} z_{ij}$ is the simulated genetic effect of the $i$-th individual for log-transformed abundance of the $k$-th OTU (Eq. (2)) and that the desired heritability for that abundance is $h_{k}^{2}$. The algorithm (see Box 1) is based on the simple observation that, given any two vectors ${\mathbf{x}}$ and ${\mathbf{y}}$, the correlation is maximum ($\rho$ ~ 1) when observations in both vectors are sorted and $\rho$ is ~ zero when they are shuffled. Therefore, there must be some order ${\mathbf{y}}_{{{\varvec{sort}}}}$ that fulfills, approximately, the constraint ${\text{cor}}({\mathbf{x}}, {\mathbf{y}}_{{{\varvec{sort}}}} ) = \rho$. For our purpose, we need to rearrange the observed abundances ${\mathbf{x}}_{k}$ such that the correlation between the rearranged ${\mathbf{x}}_{k}$ and ${{\varvec{\upgamma}}}_{k}$ is $h_{k}$, i.e. the square root of heritability for abundance of the $k$-th OTU. The detailed algorithm is provided in Box 1. As a result, with this algorithm a covariance between genome and microbiome is generated in the ‘Recursive’ and ‘Indirect ‘scenarios, mediated by Eqs. (1) and (2).

A drawback of this algorithm is that it locally breaks the covariance between abundances of different OTU. To alleviate this, we permuted all abundances that fell within the same OTU cluster. We clustered abundances using the R function hclust(dist(.), method = "ward.D2") and cut the tree in K = 500 clusters. We chose K = 500 because the first quartile of the intra-cluster average correlation was above the third quartile of the average correlation between random abundances, i.e., clusters were made up of highly correlated abundances compared to average. We also explored K = 200 but we found no difference in predictive accuracy. To verify that the shuffling algorithm did not alter the structure of the data, we show the results of the principal component analysis of the original microbiome set and a few shuffled microbiome sets in Additional file 1: Figure S2. Causative OTU were sampled from different clusters.

Box 1 Finding a permutation of vectors ${\mathbf{x}}$ and ${\mathbf{y}}$ such that the correlation between permuted vectors is a predetermined value ${\varvec{\rho}}$.

Take ${\mathbf{x}}$, ${\mathbf{y}}$**,** and $\rho$, where ${\mathbf{x}}$ and ${\mathbf{y}}$ are arbitrary uncorrelated vectors in $R^{n}$ and $0 \le \rho \le 1$ is the desired correlation. The aim is to find a permutation of ${\mathbf{y}}$ such that the correlation ${\text{cor}}({\mathbf{x}}, {\mathbf{y}}_{{{\varvec{sort}}}} ) = \rho$, approximately. The algorithm can be equally applied when ${\mathbf{x}}$ and/or ${\mathbf{y}}$ consist of integer numbers and normality is not required either. The performance of the algorithm improves as $n$ increases and when normality does hold.

1. Sort the values of ${\mathbf{x}}$ and ${\mathbf{y}}\user2{ }$ in increasing or decreasing order. The correlation ${\text{cor}}\left( {{\mathbf{x}}_{{{\varvec{sort}}}} ,{\mathbf{y}}_{{{\varvec{sort}}}} } \right) \cong 1.$
2. Generate a dummy variable ${\mathbf{z}} = {\mathbf{y}}_{{{\varvec{sort}}}} + {\mathbf{e}}\user2{ }$ where ${\mathbf{e}}$ values are sampled from ${\mathbf{e}}\user2{ }\sim N\left( {0,S_{y}^{2} \frac{{1 - \rho^{2} }}{{\rho^{2} }} } \right),$ with $S_{y}^{2}$ being the sample variance of ${\mathbf{y}}$. The correlation ${\text{cor}}\left( {{\mathbf{x}}_{{{\varvec{sort}}}} ,{\mathbf{z}}} \right)\sim \rho$.
3. Create an index vector ${\mathbf{i}}_{{\mathbf{y}}}$, which indicates how ${\mathbf{y}}_{{{\varvec{sort}}}}$ should be reordered according to the order of ${\mathbf{z}}$. This dummy index ${\mathbf{i}}_{{\mathbf{y}}} = order\left( {\mathbf{y}} \right)\left[ {order\left( {\mathbf{z}} \right)} \right]$ contains the order of ${\mathbf{y}}$ when values are back-sorted according to the order of ${\mathbf{z}}$.
4. Reorder ${\mathbf{i}}_{{\mathbf{y}}} = {\mathbf{i}}_{{\mathbf{y}}} \left[ {{\text{rank}}\left( {\mathbf{x}} \right)} \right]$ to match the index with positions ${\mathbf{y}}_{{{\varvec{sort}}}}$ in the original vector ${\mathbf{x}}$. This is needed since ${\mathbf{x}}$ remains unchanged and only ${\mathbf{y}}$ is permuted.
5. The correlation $${\text{cor}}\left( {{\mathbf{x}},{\mathbf{y}}\left[ {{\mathbf{i}}_{{\mathbf{y}}} } \right]} \right) \cong \rho$$.

### Parameter fitting

Little is known on the number of OTU that influence a given phenotype and on how many of those are partly inherited. For that reason, we chose some extreme but ‘educated’ values for each of the five scenarios depicted in Fig. 1. We considered $r^{2} = h_{g}^{2} + h_{b}^{2}$, where $h_{g}^{2}$ and $h_{b}^{2}$ are the heritability and microbiability, respectively; $r^{2}$ = 0.25 is grossly the value reported by Difford [4] with N = 750, whereas values closer to $r^{2}$ = 0.50 were reported by Wallace et al. [13] for some farms. In general, increasing $r^{2}$ attempts to mimic the effect of increasing sample size. We assumed $h_{g}^{2} = h_{b}^{2}$ for the ‘Joint’ and ‘Recursive’ scenarios, as approximately reported by Difford et al. [4] and Camarinha-Silva et al. [16]. The number of QTN was somewhat arbitrary and set to either 100 or 500, but the specific number of loci should not affect the results much. Barton et al. [47] showed theoretically that most properties of the infinitesimal model hold as the number of QTN increases even modestly (N > 20).

Numerous empirical and theoretical works have shown that genetic effects of QTN on phenotype are not uniformly distributed and can be approximated by a gamma-like distribution [48, 49]. Thus, here we sampled direct genetic effects $\alpha \sim {\Gamma }$ (shape = 0.2, scale = 5), as suggested by Caballero et al. [50] and also used by us previously [51].

Much less is known on the number of causative OTU ($N_{OTU}$), although we can presume that $N_{OTU}$ should be smaller than the number of QTN. For instance, Duvallet et al. [36] found in a large meta-analysis that the human diseases studied were affected by, on average, 10 to 15 changes in abundances at the genus level. Here, we considered $N_{OTU}$ = 25 (0.6% of all OTU), although we also evaluated $N_{OTU}$ = 10, 100, and 250 (Table 2). Similarly, for the ‘Recursive’ and ‘Indirect’ scenarios, we took the extreme scenario where all causative OTU are genetically determined, i.e., $N_{OTU} = N_{OTU\left( g \right)}$. The genetic effects $\beta$ on abundances (Eq. (2)) were sampled from the same distribution $\beta \sim {\Gamma }$(shape = 0.2, scale = 5) as the direct genetic effects $\alpha$. Much less is known about the distribution of the effects of abundances, $\omega$, on phenotype (Eq. (1)). We took as proxy the distribution of estimates of regression coefficients of methane emission on abundances reported by Difford et al. [4] in their supplementary information S4, which can be approximated by ~ ${\Gamma }$(shape = 1.4, scale = 3.8). Additional file 1: Figure S3 compares the distributions of QTN and OTU effects and their fit to the empirical data of Difford et al. [4]. This model predicts that the variance of the effects of OTU on phenotype is on average wider and larger than that of QTN. Although, at this point, this is speculative, it is sensible to assume that only a few taxa have a sizeable influence on a phenotype such as methane emission.

### Data analysis

It was not evident which predictive algorithm would work best for the complex scenarios simulated here, although results from the literature show that no approach is optimal for all cases. Here, we compared the Bayes C algorithm [20] and Bayesian RKHS regression, which is equivalent to GBLUP [38], to assess prediction performance and reliability of parameter estimates. Both approaches represent extreme parameterizations in terms of priors applied and were implemented in the BGLR R package [52]. To assess predictive accuracy, 75 (10%) phenotypes were randomly removed and predicted with the fitted model using the remaining data. The correlation between observed and predicted phenotypes was used as a measure of predictive accuracy.

The generic linear model used was:

3 $${\mathbf{y}} = {\mathbf{Zg}} + {\mathbf{Wb}} + {\mathbf{i}} + {\mathbf{e}},$$

where ${\mathbf{y}}$ is the vector of the simulated phenotypes, ${\mathbf{g}}$ is the vector of SNP effects, ${\mathbf{Z}}$ is a matrix of the observed genotypes for the 33 k SNPs, ${\mathbf{b}}$ is the vector of the effects of log-transformed abundance of OTU, ${\mathbf{W}}$ is a matrix with all $n_{b}$ = 4018 log-transformed abundances for the $n$ = 750 individuals, ${\mathbf{i}}$ contains the interaction between $g$ and $b$, and ${\mathbf{e}}$ is a vector of residuals. Prior to the analyses, phenotypes, abundances and genotypic values were standardized to a mean of zero and a SD of 1. For Bayesian RKHS, variance–covariance structures were specified to be ${\text{Var}}\left( {\mathbf{g}} \right) = {\mathbf{G}}$, ${\text{Var}}\left( {\mathbf{b}} \right) = {\mathbf{B}}$, and ${\text{Var}}\left( {\mathbf{i}} \right) = {\mathbf{G}} \circ {\mathbf{B}}$, with ${\mathbf{G}} = {\mathbf{ZZ^{\prime}}}/n$, ${\mathbf{B}} = {\mathbf{WW^{\prime}}}/n_{b}$, and $\circ$ denotes the direct product between matrices. The term ${\mathbf{i}}$ is intended to capture any variance due to the interaction between genome and microbiome, similar to the additive x additive epistatic variance–covariance structure usually being obtained from ${\mathbf{G}} \circ {\mathbf{G}}$ [53, 54]. For priors, we used the default values in the BGLR software.

In Bayes C, we did not include an interaction explicitly but, instead, computed a covariance between estimates $b$ and *g*, as detailed below in Eq. 3. As priors ${\uppi }$ for the probability of SNPs or abundances to enter the Bayes C model, we used ${\uppi }\sim {\text{Beta}}\left( {{\text{p}}_{0} = 5,{\uppi }_{0} = 0.001} \right)$, which has expectation ${\uppi }_{0}$ and variance ${\uppi }_{0} \left( {1 - {\uppi }_{0} } \right)/{\text{p}}_{0} + 1)$. We also considered a much more liberal flat prior for ${\uppi }\sim {\text{Beta}}\left( {{\text{p}}_{0} = 2,{\uppi }_{0} = 0.01} \right)$, but we did not observe strong differences (see Additional file 1: Figure S1). Unlike GBLUP, ‘heritability’ is not explicitly defined in a Bayes C framework but, here, we used the proposal by [52] (https://github.com/gdlc/BGLR-R/blob/master/inst/md/heritability.md) to estimate heritability and microbiability. In short, at each iteration $i$ of the MCMC, the algorithm samples the effects of the SNPs and OTU:

$${\mathbf{u}}^{\left( i \right)} = {\mathbf{Z}}{ }{\hat{\mathbf{g}}}^{\left( i \right)} ,$$

$${\mathbf{v}}^{\left( i \right)} = {\mathbf{W}}{ }{\hat{\mathbf{b}}}^{\left( i \right)} ,$$

where ${\mathbf{u}}^{\left( i \right)}$ and ${\mathbf{v}}^{\left( i \right)}$ are genome and microbiome effects at the $i$-th iteration for the set of individuals, respectively, and ${\hat{\mathbf{g}}}^{\left( i \right)}$ and ${\hat{\mathbf{b}}}^{\left( i \right)}$ are the sampled effects of the SNPs and OTU abundances; therefore, ${\text{Var}}({\mathbf{u}}^{\left( i \right)} )/{\text{Var}}\left( {\mathbf{y}} \right)$ and ${\text{Var}}({\mathbf{v}}^{\left( i \right)} )/{\text{Var}}\left( {\mathbf{y}} \right)$ are the sampled heritability and microbiability in the $i$-th iterate, from which posterior means were estimated by averaging over iterations. For Bayes *Cgb*, we also computed the sampled absolute covariance between ${\mathbf{u}}$ and ${\mathbf{v}}$ for each iteration $i$, i.e.:

4 $$\left| {Cov\left( {{\mathbf{u}},{\mathbf{y}}} \right)} \right| = \frac{{\mathop \sum \nolimits_{i = 1}^{Niter} \left| {cov\left( {{\varvec{u}}^{i} ,{\varvec{v}}^{i} } \right)} \right|}}{{ Var\left( {\varvec{y}} \right) Niter}}^{{}}$$

To assess how likely it is to identify causative OTU in Bayes C, we computed the probability of a given OTU to enter the model over MCMC iterations. We ran a GWAS of abundances (${\mathbf{x}}_{k}$, $k = 1$, $N_{OTU}$) on SNP genotypes (${\mathbf{z}}_{j}$, $j = 1$, $N_{SNP}$) using the R function lm(${\mathbf{x}}_{k} \sim {\mathbf{z}}_{j}$) and computed the association P-value of both causative QTN, i.e., those that were simulated to affect abundances, and of neutral SNPs. This was done in the ‘Recursive’ scenario only, in which we also computed the heritabilities of all abundance levels using the RKHS model. Weakly informative priors for variances were used in this case to mimic a REML-like estimator.

We ran RKHS and Bayes C with complete models, i.e., including genome, microbiome and their interaction, and with partial models that considered only microbiome or genome information (Table 3). This was done to study confounding and to determine whether part of the variance in microbiome abundances was captured by the genome when the microbiome was partly heritable (‘Indirect’ and ‘Recursive’ scenarios). In total, 50k iterations, including 500 burn-in iterations and thinning every 5, were run for both the RKHS or Bayes C chains; a plot of the variances against iteration number indicated that convergence was attained with this number of iterations (see Additional file 1: Figure S2).

**Table 3:** Statistical models used to analyze the data

Method	Abbreviation	Effects fitted		
		${\varvec{g}}$	${\varvec{b}}$	${\varvec{g}} \times {\varvec{b}}$
Bayesian RKHS (GBLUP)	*Rgbx*	x	x	x
	*Rgb*	x	x	-
	*Rg*	x	-	-
	*Rb*	-	x	-
Bayes C	*Cgb*	x	x	-
	*Cg*	x	-	-
	*Cb*	-	x	-

Effects included can be $g$ (SNP genotypes), $b$ (OTU abundances) and their interaction $g \times b$

## Results

### How useful can the microbiome be for prediction of complex traits?

This logically depends on how much phenotypic variance is jointly explained by the genome ($h^{2}$) and the microbiome ($b^{2}$), but also on how efficiently methods capture the relationship between the microbiome and the phenotype, and on how stable the microbiome is. It should be noted that prediction accuracy is conditionally independent of heritability of the microbiome itself, i.e., *given* the observed abundances B and observed genotypes G, it does not matter whether the biological processes that generate B are affected by G. In other words, for a constant $r^{2} = h^{2} + b^{2}$, prediction should not be affected by whether the ‘Joint’ or ‘Recursive’ scenarios hold. Implications for genetic improvement, however, could be dramatically different. Breeding schemes that target the microbiome could be designed provided the ‘Recursive’ scenario holds but make no sense under the ‘Joint’ scenario.

**Fig. 3:**
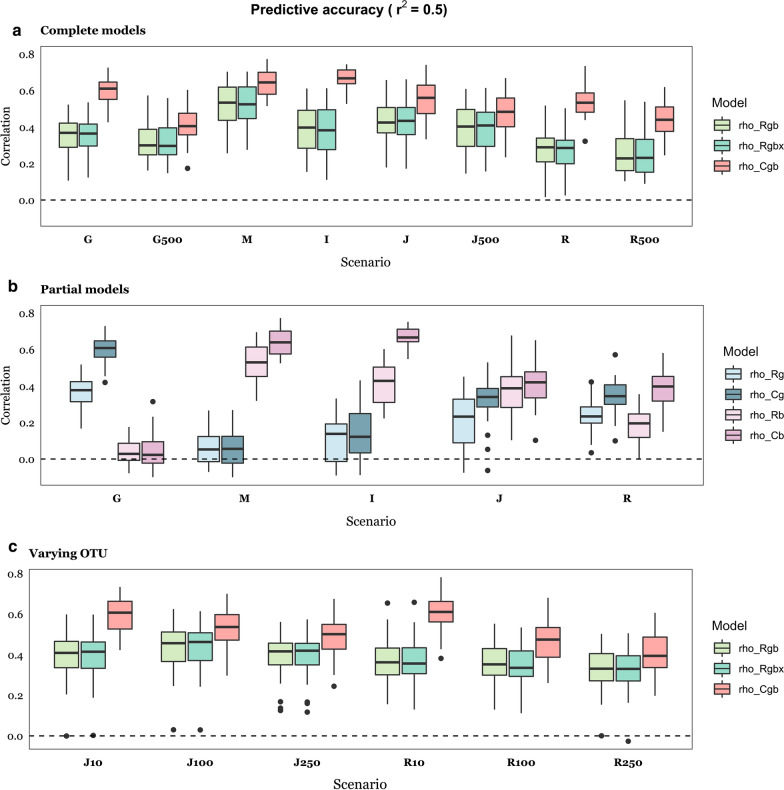
Predictive accuracy with *r*^2^ = 0.50, computed as correlation between predicted and observed phenotypes across causal scenarios (Fig. 1), for each of the RKHS (Bayes C) models. *Rgb (Cgb*) considers microbiome and genome data; *Rgbx* includes genome and microbiome data and their interaction; *Rg (Cg*) includes genome data only; *Rb (Cb*) includes microbiome data only. **a** Complete models; **b** Partial models, **c** Effect of varying the number of causative OTU. Details of scenarios are in Tables 1 and 2: G: Genome; M: Microbiome; I: Indirect; J: Joint; R: Recursive. G500, J500 and R500 means 500 causative SNPs; J10 and R10, 10 causative OTU; J100 and R100, 100 causative OTU; J250 and R250, 250 causative OTU. Results are an average of 30 replicates per case

**Fig. 4:**
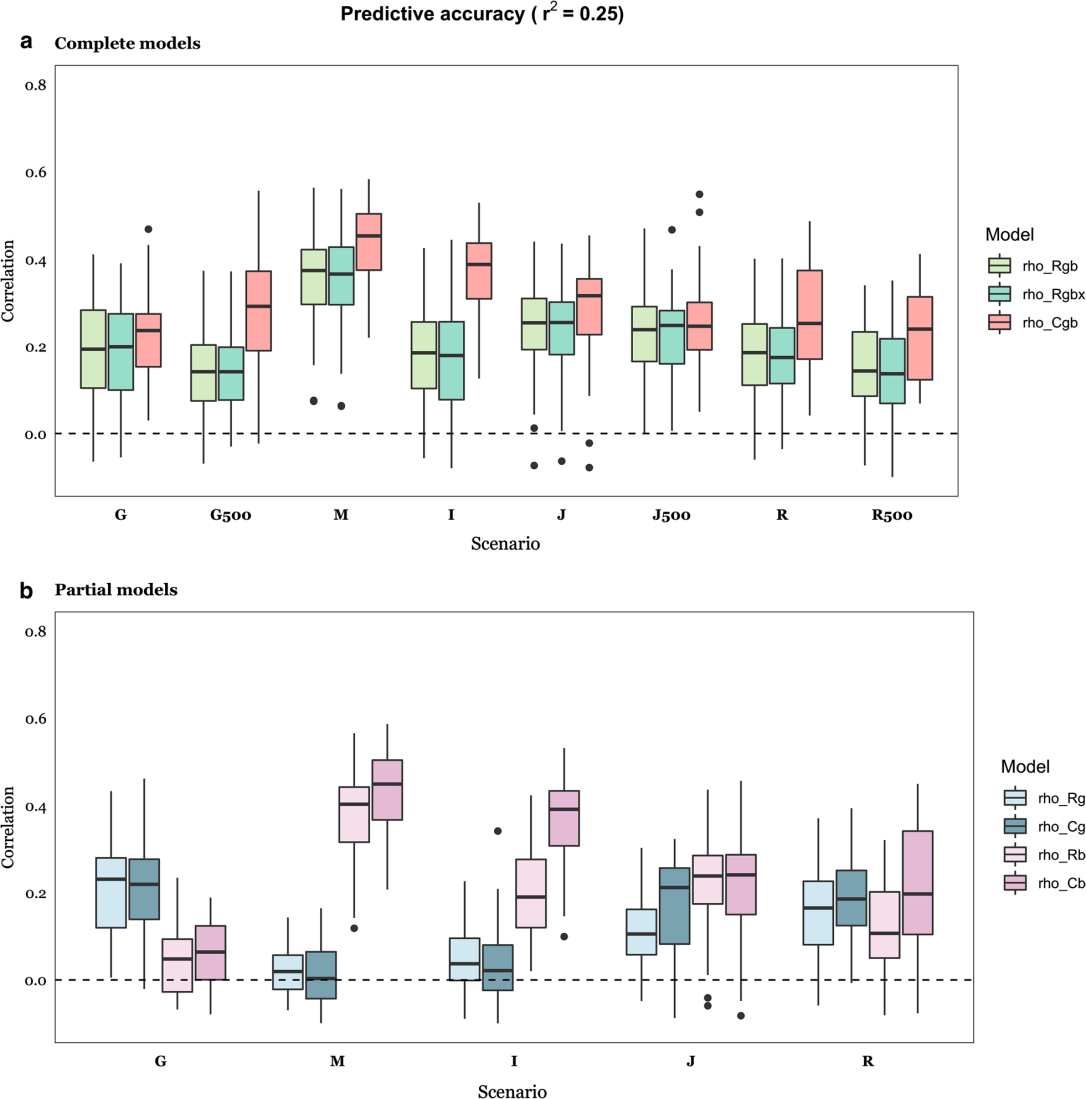
Predictive accuracy with ${\text{r}}^{2}$ = 0.25, computed as correlation between predicted and observed phenotypes across causal scenarios (Fig. 1), for each of the RKHS (Bayes C) models. *Rgb* (*Cgb*) considers microbiome and genome data; *Rgbx* includes genome and microbiome data and their interaction; *Rg (Cg*) includes genome data only; *Rb (Cb*) includes microbiome data only. **a** Complete models; **b** Partial models. Details of scenarios are in Tables 1 and 2: G: Genome scenario; M: Microbiome; I: Indirect; J: Joint; R: Recursive. G500, J500 and R500 means 500 causative SNPs. Results are an average of 30 replicates per case

We compared the predictive performance of the Bayesian RKHS (GBLUP-like approach) and Bayes C [20] approaches when both genome and microbiome data were included in the model (*Rgb* and *Cgb*), including an interaction term between $g$ and $b$ (*Rgbx*), or only genome data (*Rg, Cg*), or only microbiome data (*Rb, Cb*). For details of all models, see Table 3. First, we verified that the null model, i.e., when phenotypes were permuted relative to genotypes and abundances, did not result in false predictive accuracies (see Additional file 1: Figure S3A). Figures 3 and 4 show the predictive accuracies for the two $r^{2}$ values considered, 0.50 and 0.25, respectively. In the case of $r^{2}$ = 0.50, we also explored the influence of varying the number of causative OTU (Table 2).

Overall, Bayes C showed better performance than RKHS, but it was more sensitive to an increase in the number of QTN or of causative OTU (Fig. 3c). Importantly, adding a term for the interaction between $g$ and $b$ did not improve the predictive performance of RKHS, even in the ‘Recursive’ scenario (*R*). The full models *Rgb* and *Cgb* were much better than the partial models (*Rg, Rb, Cg, Cb*) when both $h^{2}$ and $b^{2}$ were larger than zero, as expected, i.e., for the ‘Joint’ and ‘Recursive’ scenarios (compare Fig. 3a vs. b and Fig. 4a vs. b). In these scenarios, using both sources of variation improved prediction compared to using only genome or microbiome data, especially when using Bayes C. Importantly, the predictive accuracy was slightly lower for the ‘Joint’ and ‘Recursive’ scenarios than for the ‘Microbiome’ or ‘Genome’ scenarios. This indicates that the predictive accuracy does not only depend on total $r^{2}$, but also on how this variance is split between genome and microbiome. Although this likely occurs because of the larger noise in the ‘Recursive’ or ‘Joint’ scenarios than in the ‘Microbiome’ or ‘Genome’ scenarios, it also suggests that our strategy of analysis may not be optimal and that there is room to develop more efficient tools, especially for the ‘Recursive’ scenario. It should be noted that the variance of prediction was larger for the ‘Recursive’ than for the ‘Joint’ scenario for $r^{2}$ = 0.25, i.e., heritability of abundances may be an additional source of noise. This effect was less pronounced as $r^{2}$ increased.

We observed that the predictions were better when only the microbiome influenced the phenotype than when the genome was the only source of variation, a phenomenon also observed with real data [13, 16, 24]. In this simulation, this likely occurred because the number of causative effects and of input variables (SNPs vs. OTU) was smaller for the ‘Microbiome’ or ‘Indirect’ scenarios than for the ‘Genome’ scenario. In fact, we observed a consistent negative correlation between the number of causative OTU and the predictive accuracy for both the ‘Joint’ and ‘Recursive’ scenarios (Fig. 3c). The number of QTN also adversely affected prediction performance but mainly with Bayes C, whereas RKHS was not largely affected (see especially scenario G vs. G500 for $h^{2}$ = 0.5 in Fig. 3a).

Taken together, our results suggest that predictive accuracy could be increased by ~ 50% when considering microbiome in addition to genome data, provided the microbiability is of the same order as the heritability (Fig. 3). This is probably an upper, optimistic limit, since it will be difficult to have microbiome data collected homogeneously over time and in different locations. While individuals can be genotyped at birth, the microbiome during early life stages may not be representative of that at adult or later stages. For instance, Maltecca et al. [55] showed that early life microbiota is not a good proxy for carcass composition in pigs, whereas later life microbiota is more strongly associated.

We observed a roughly two-fold increase in predictive accuracy when heritability was doubled for the ‘Genome’, ‘Joint’ and ‘Recursive’ scenarios, and a 50% increase for the ‘Microbiome’ and ‘Indirect’ scenarios (Fig. 3 vs. Fig. 4).

### Are microbiability estimates reliable?

Reliable parameter estimates are needed to optimize the design of breeding schemes, management practices or microbiome-wide association studies (MWAS [56]). They are also needed to understand the biology that underlies the interaction between microbiome and complex phenotypes. To date, microbiability has usually been estimated using ‘standard’ linear methods, e.g., [4, 11, 32], much as we have done here. Thus, it is of interest to know how accurate these estimates are.

**Fig. 5:**
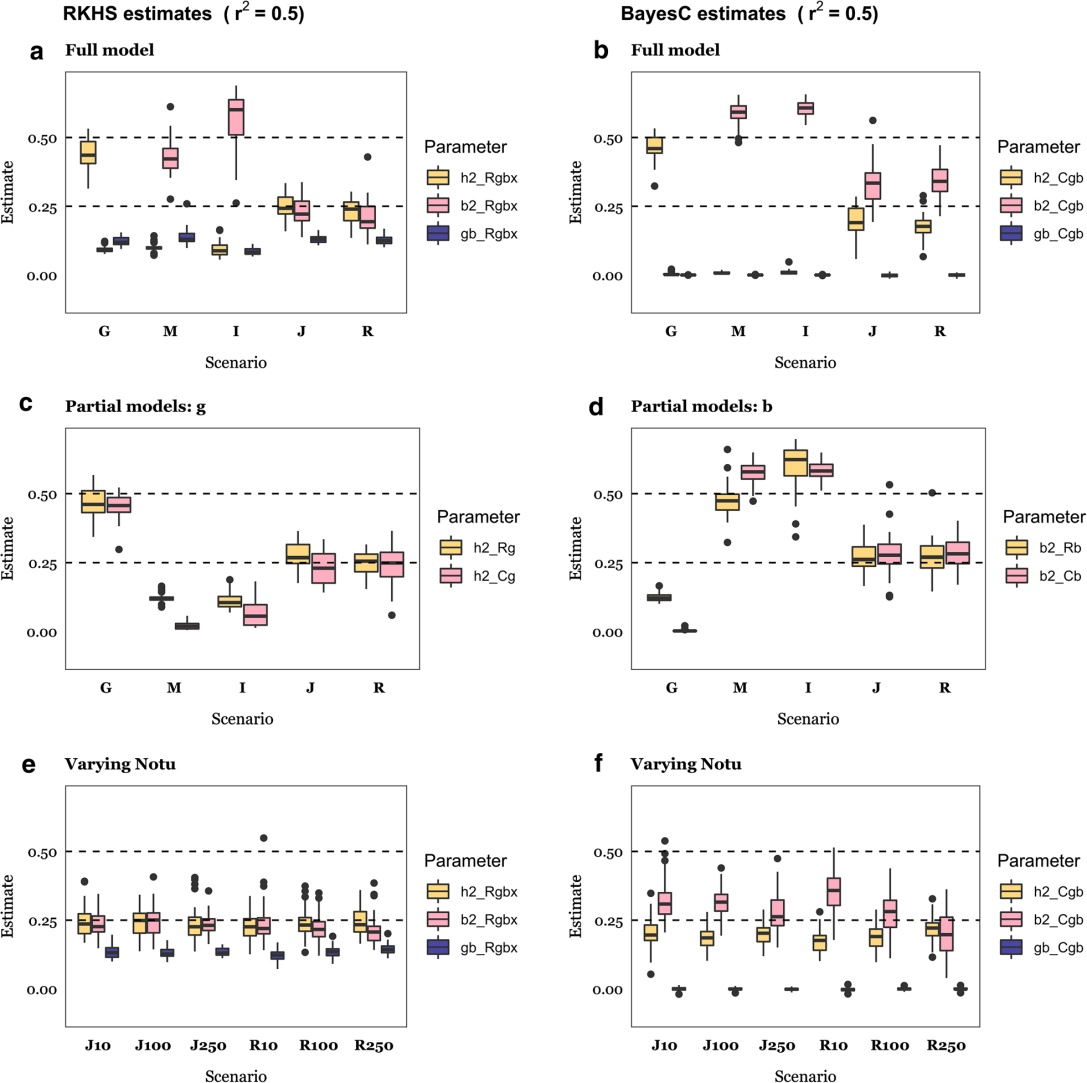
Parameter estimates for $r^{2}$ = 0.50*.* Estimates of heritability ($h^{2}$), microbiability ($b^{2}$), and the correlation between the genome and microbiome ($gb$) for each of the RKHS (Bayes C) analysis models: model *Rgbx* includes microbiome and genome data and their interaction; *Cgb* includes genome and microbiome data; *Rg (Cg*) includes genome data only, and *Rb* (*Cb*) includes microbiome data only. Details of simulation scenarios are in Tables 1 and 2: G, Genome scenario; M, Microbiome; I, Indirect; J, Joint; R, Recursive; G500, J500 and R500 mean 500 causative SNPs; J10 and R10, 10 causative OTU; J100 and R100, 100 causative OTU; J250 and R250, 250 causative OTU. The horizontal dashed lines indicate simulated $h^{2}$ or $b^{2}$ parameter values (0.25, 0.5 depending on the scenario). Results are an average of 30 replicates

**Fig. 6:**
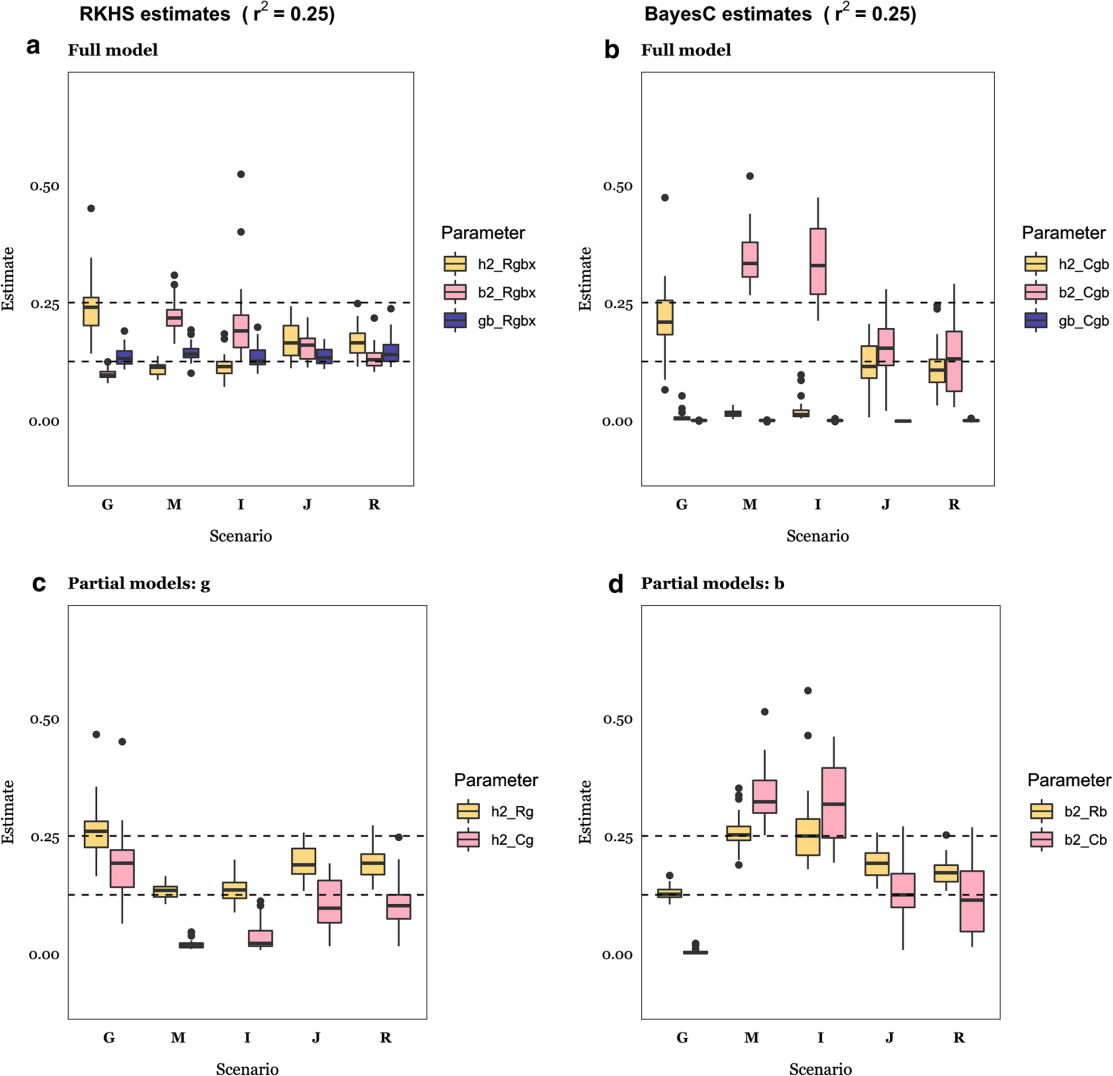
Parameter estimates for $r^{2}$ = 0.25. Estimates of heritability ($h^{2}$), microbiability ($b^{2}$), and the correlation between the genome and microbiome ($gb$) for each of the RKHS (Bayes C) analysis models: model *Rgbx* includes microbiome and genome data, and their interaction; *Cgb* includes genome and microbiome data; *Rg (Cg*) includes genome data only, and *Rb (Cb*) includes microbiome data only). Details of simulation scenarios are in Table 1: G: Genome scenario; M: Microbiome; I: Indirect; J: Joint; R: Recursive. The horizontal dashed lines indicate simulated $h^{2}$ or $b^{2}$ parameter values (0.125, 0.25 depending on the scenario). Results are an average of 30 replicates

Figures 5 ($r^{2}$ = 0.50) and 6 ($r^{2}$ = 0.25) show estimates of the variance components for each of the scenarios and model analyses from Tables 1, 2, 3. Bayes *Cgb* allows us to assess whether $h^{2}$ and/or $b^{2}$ differ from zero: the microbiability estimate was on average near zero when the data were simulated according to the ‘Genome’ scenario and the heritability estimate was zero when the ‘Indirect’ or ‘Microbiome’ scenarios hold, as expected (Figs. 5b and 6b). Similarly, estimates of both $h^{2}$ and $b^{2}$ were near zero when the null scenario held (see Additional file 1: Figure S3B). However, the behavior of estimates obtained with RKHS was different, as variance ratios are a priori bound between 0 and 1: average estimates of $h^{2}$ and $b^{2}$ were small yet non-zero when simulated values were zero. Comparing Fig. 5a vs. Fig. 5b for estimates of $h^{2}$ *(*$b^{2}$) in the ‘Microbiome’ (‘Genome’) scenarios, estimates are clearly zero with Bayes C, as expected, but not with RKHS. The effect of prior information is much stronger for low $r^{2}$, resulting in RKHS estimates that should be zero to be more biased (Fig. 6a).

It is interesting to observe that the heritability and microbiability estimates obtained with RKHS were less biased than those obtained with the Bayes C algorithm, except when the true parameter is zero. This was more apparent for the ‘Joint’ and ‘Recursive’ scenarios and $r^{2}$ = 0.5, as observed in the comparison between Fig. 5a and Fig. 5b. With Bayes C, an overestimation of $b^{2}$ is evident, regardless of the simulated value of $r^{2}$. For the ‘Joint’ and ‘Recursive’ scenarios, the upward bias in the estimate of $b^{2}$ was accompanied by an underestimation of $h^{2}$, which indicates that variance estimates were confounded when using the Bayes *Cgb* model (Fig. 5b). However, this bias decreased when the number of causative OTU increased. For instance, the bias in the $b^{2}$ estimate was ~ 40% when $N_{OTU}$ = 10 but reduced to ~ 10% with $N_{OTU}$ = 250 (Fig. 5c). In contrast, estimates obtained with RKHS were remarkably robust to varying $N_{OTU}$ (Fig. 5d vs. e). Therefore, it is likely that the presence of a few causative OTU, but of large effect, combined with the presence of highly leptokurtic abundance distributions, may result in biased parameter estimates when using Bayes C. It should be noted, in turn, that estimates obtained with RKHS were inflated when they were actually zero, i.e., when the model was overparameterized. This should be considered when interpreting microbiability estimates from real data. For instance, Difford et al. [4] report estimates of $h^{2}$ = 0.21 and $b^{2}$ = 0.13 (N = 750) and found that G and B are independent. Assuming the number of causative OTU is small compared to the number of SNPs with an effect on abundances (QTN), our simulation results suggest that the estimate of $b^{2}$ reported by Difford et al. [2] might be inflated. If this is true, the actual contribution by the microbiome might be too small to improve prediction over that obtained from using only genome data. Although Difford et al. [4] focused on inference rather than on prediction, they reported that no bacteria genera were significantly associated with methane emissions. Other authors have reported multiple microbial associations with methane emissions, including members of bacterial, archaeal, fungal, and protozoan communities, e.g., [13, 30, 57–59].

For comparison, panels c and d in Figs. 5 and 6 show estimates that were obtained when only genome or microbiome information was used. The most noticeable outcome is that bias in estimates of $b^{2}$ was somewhat reduced relative to that found with Bayes *Cgb*, which again indicates that some confounding between $b^{2}$ and $h^{2} {\text{ occurred}}$. In general, bias was lower when $r^{2}$ was greater but did not vanish.

### Can the underlying biological scenario and causative OTU be recovered?

An important goal of many experiments is to dissect the biological basis of the interactions between the microbiome and the genome, even if this is not strictly needed for prediction. So far, our simulations suggest that standard statistical methods can be used to quantify—with some bias—the contribution of microbiability to the phenotypic variance. It also appears possible to distinguish which of the ‘Microbiome’ or ‘Genome’ scenarios fit a dataset best. Similarly, it appears possible to assess whether both G and B contribute to the phenotypic variance, i.e., whether the ‘Recursive’ or ‘Joint’ scenarios are plausible. A question, however, is whether it is possible to distinguish between the ‘Joint’ and ‘Recursive’ scenarios, i.e., whether the data can indicate which of the ‘Indirect’ or ‘Microbiome’ scenarios is more plausible, if either. Furthermore, can causative OTU be identified? These are far more difficult questions to answer than assessing prediction performance or estimating microbiability. When the variance component estimates obtained under the ‘Joint’ and ‘Recursive’ scenarios are compared (Figs. 5 and 6), they appear to be nearly identical for the same $r^{2}$. The two scenarios differ in that at least some causative OTU abundances are under partial genetic control in the ‘Recursive’ scenario. Thus, the ‘Recursive’ scenario should result in a covariance between G and B. For the RKHS modeling, we studied whether this covariance could be partly captured by adding an interaction factor ${\mathbf{g}} \circ {\mathbf{b}}$ (Eq. 3) much as imperfect disequilibrium can generate ‘phantom’ epistasis [60]. However, the interaction estimates were not found to be greater in the ‘Recursive’ than in the ‘Joint’ or ‘Microbiome’ scenarios when no interaction was simulated (Figs. 5a and 6a). As for Bayes C, we investigated whether the two scenarios could be distinguished by analyzing the covariance ${\text{cov}}({\mathbf{u}}^{\left( i \right)} ,{ }{\mathbf{v}}^{\left( i \right)} )/{\text{var}}\left( {\mathbf{y}} \right)$ (see Methods). Again, these estimates were close to zero regardless of the simulated scenario (Figs. 5b and 6b).

**Fig. 7:**
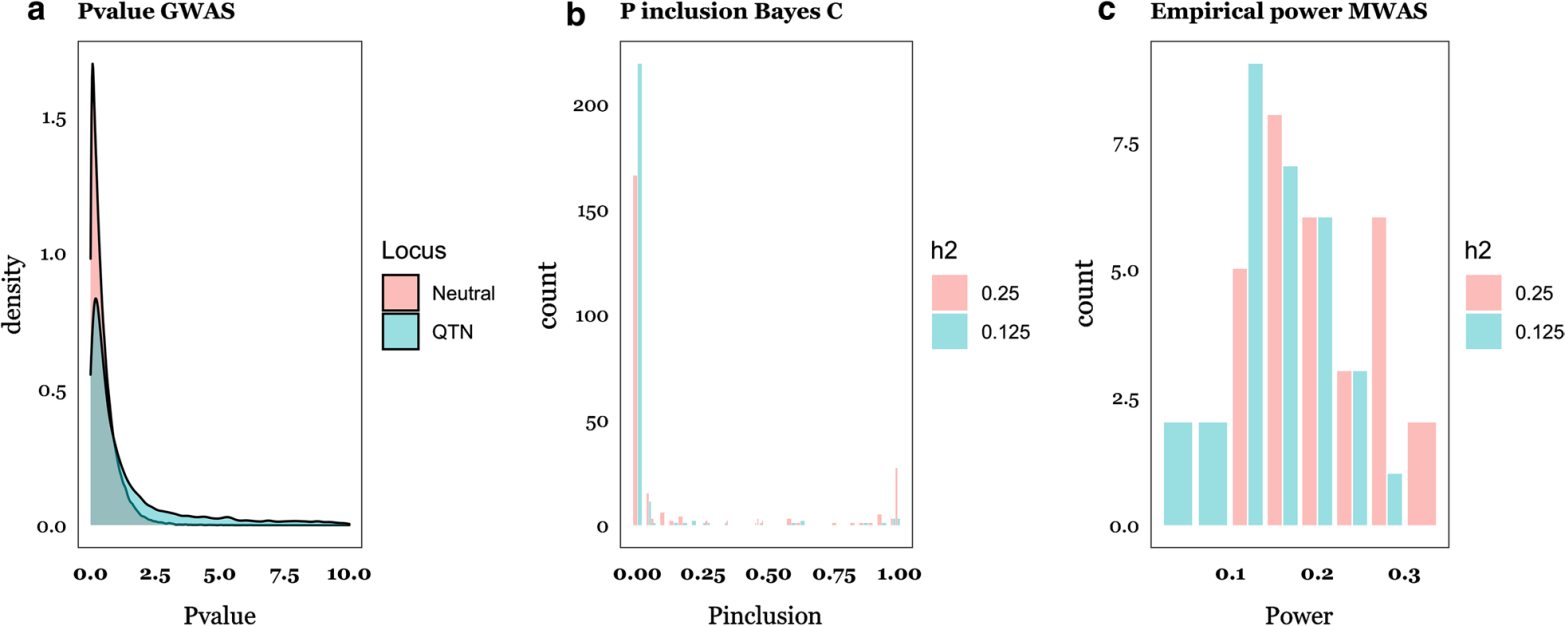
Power in genome- (GWAS) and microbiome- (MWAS) wide association studies. **a** Distribution of -log10 P-values of a GWAS of abundances. **b** Probability of inclusion in the Bayes *Cgb* model of causative OTU for the two levels of microbiability considered. **c** Power of identifying a causative OTU computed as the probability of exceeding the 95% threshold of the empirical distribution of P-values in an MWAS for the Recursive scenario

An alternative approach to infer whether the ‘Recursive’ scenario holds or not is to run a genome-wide association study (GWAS) for each OTU abundance. If we identify significant SNPs for OTU that are likely to influence the phenotype y, we could conclude that the ‘Recursive’ scenario is plausible. Unfortunately, this analysis can be doomed by the large number of tests to be performed, i.e., $N_{OTU} \times N_{SNP}$. To illustrate the caveats of GWAS on abundances, Fig. 7a shows the distribution of -log10 P-values of neutral SNPs vs. SNPs with an effect on abundances. Taking the 5% empirical threshold of the neutral P-value distribution to declare an association, simulations suggest that the P-values of only ~ 3% of the causative SNPs will be above that threshold, i.e., approximately what is expected by chance. These P-values depend of course on the actual number of causative SNPs and on abundance heritabilities, but most of the evidence to date points to a weak relationship between the genome and the microbiome [27]. It will be very difficult to identify causative SNPs for abundance using GWAS information alone [11, 25].

Another question of interest is what proportion of the OTU that affect the phenotype we can expect to discover. One option is to count the frequency with which a given OTU enters the Bayes C model during sampling. Figure 7b shows the probability of including a causative OTU in the Bayes C sampling chain, which ranged from ~ 5% ($b^{2}$ = 0.125) to ~ 20% ($b^{2}$ = 0.25). On average, about 50 ($b^{2}$ = 0.25) and 30% ($b^{2}$ = 0.125) of the causative OTU were among the 5% most frequently included OTU in the Bayes C chain. Nevertheless, since the number of causative OTU was 25, the rate of false positives was high. We can conjecture that only a few causative OTU are likely to be identified in medium-sized experiments, such as simulated here.

An alternative approach is a microbiome-wide association study (MWAS), i.e., to perform a linear regression of the phenotype on each of the OTU abundances and then select the significant OTU as potential causative OTU [4]. Figure 7c shows the average power, defined as the percentage of true causative OTU among the 5% most significant OTU. In the ‘Recursive’ scenario, power was ~ 15 and ~ 20% for $b^{2}$ = 0.125 and 0.25, respectively. Again, this is not too satisfactory since we expect a high fraction of false positives. In this scenario, it is perhaps more useful to consider probabilities of inclusion in the Bayes C chain rather than P-values from linear regression since the former are the result of a joint analysis of all OTU and can be used directly for prediction.

**Fig. 8:**
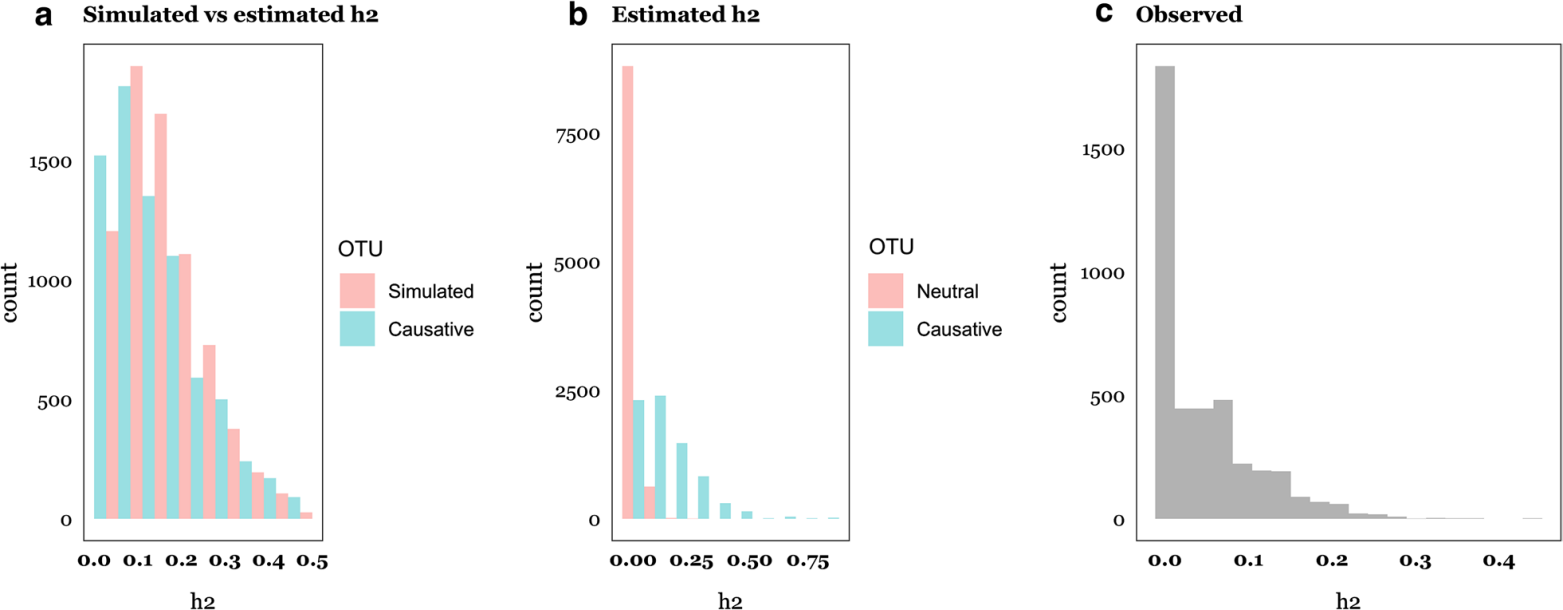
Simulated, estimated and observed abundance heritabilities. **a** Distributions of simulated and GBLUP estimated heritabilities of abundance for causative OTU in the Recursive scenario. **b** Distribution of GBLUP estimated heritabilities of abundance for neutral and causative OTU in the Recursive scenario. **c** Distribution of estimates of heritabilities of OTU abundance reported by Difford et al. [4]

**Fig. 9:**
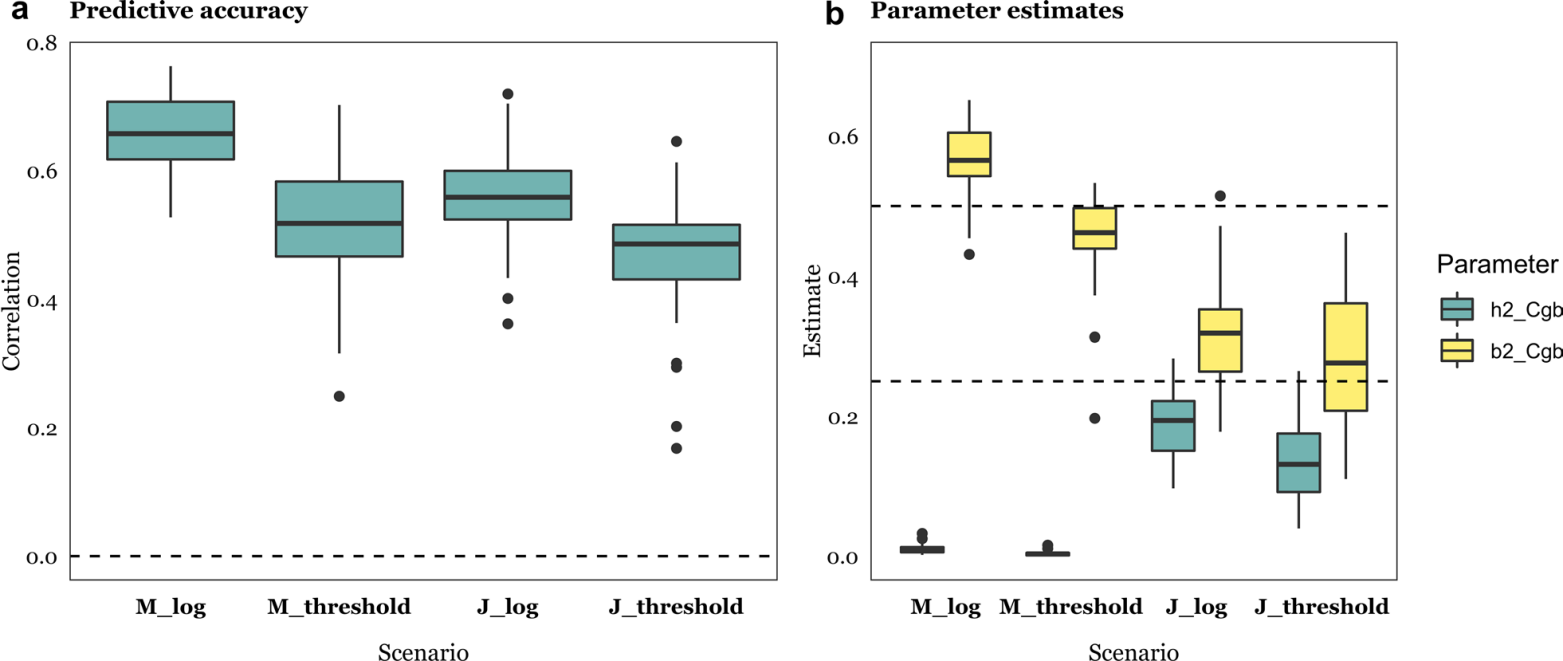
Comparison of multiplicative (log) and threshold ‘Microbiome’ (M) and ‘Joint’ (J) scenarios ($r^{2}$ = 0.5) using Bayes Cgb. **a** Predictive accuracy, computed as the correlation between predicted and observed phenotypes; and **b** Estimates of heritability ($h^{2}$) and microbiability ($b^{2}$). Results are the average of 30 replicates. Scenarios M and J as specified in Table 1; the log transformation results are shown for completeness and are the same as in Figs. 3 and 5. Data are average of 30 replicates

Finally, we investigated the pattern of abundance heritabilities. Figure 8a shows the simulated heritabilities for the causative inherited OTU, which approximately followed a gamma distribution, as well as the estimated heritabilities for the causative OTU in the ‘Recursive’ scenario. The two distributions were rather similar, although the estimates were slightly shrunk towards zero, a consequence of using a REML-like prior. Of course, a problem with real data is that we do not know which OTU are inherited, and which are not, and the true distribution of OTU heritability estimates will be a mixture due to heritable and not heritable abundances. Figure 8b illustrates the distributions of heritability estimates of neutral (non-inherited) and causative inherited OTU. In Fig. 8b, we mixed 1.7 neutral OTU per causative OTU, which is arbitrary since we do not know the actual number of OTU under genetic control, but the resulting mixture is similar to the distribution of heritabilities observed by Difford et al. [4] (Fig. 8c). If the distributions in Fig. 8b were representative of the true state of nature, this would suggest that about 1/(1 + 1.7) ~ 40% of rumen OTU could be subject to additive genetic variance in the experiment reported by Difford et al. [4] (Fig. 9).

## Discussion

### Given the uncertainty with the true scenario, a flexible simulation approach is proposed

Figure 1 represents highly simplified relationships between the genome, microbiome, and phenotype. These scenarios cover a wide range of possible interactions that are not exhaustive. For example, we did not consider the case of a phenotype having a causative effect on abundances, i.e. the ‘reversed’ Microbiome scenario. The scenarios chosen are nevertheless important to interpret empirical data and can help to identify limiting factors for prediction of complex traits. Furthermore, provided that a good fit is found, they will help the design of experiments that combine microbiome and genetic data. We chose combinations of parameters that represent extreme case scenarios and found that the results were, qualitatively, robust to the choice of parameters such as $r^{2}$. However, a parameter that can be relevant is the number of causative microbiome taxa, i.e., those with an effect on the phenotype, which appears to affect the bias of microbiability estimates when Bayes C is used (Fig. 5).

In this study, we proposed a new simulation procedure that addresses some important challenges. First, the algorithm avoids the need for actual phenotype simulation by using real genotype and abundance data. Although we concede that this procedure may limit the generality of the study, e.g., in terms of data size or abundance patterns, we believe that the advantages of using real data are numerous, since no simulation procedure can accommodate all known and unknown subtleties of the highly dimensional distributions at hand. Second, we developed a permutation procedure (see Box 1) that allows linking previously uncorrelated data to fit a target correlation value. Also, by only permuting OTU within clusters, we minimized disruption of the overall covariance structure (see Additional file 1: Figure S2). It should be noted that an OTU or SNP does not need to be causative to be useful for prediction and our simulation strategy allows existing correlations between abundances or among SNPs (i.e. linkage disequilibrium) to be maintained to mimic this fact.

For this work, we used real rumen microbiome and genomic data from Holstein dairy cattle. Methane emissions and the rumen ecosystem make a fundamental area of microbiome research, but we believe our results can be applied to other environments and species as well, including humans, provided that similar generative parameters hold. We considered a range of values for heritability and microbiability, from 0 to 0.50, which covers most of the estimates reported in the literature across species, e.g., [3, 13, 30, 61]. Human studies tend to use a larger number of SNPs than the high-density cattle genotyping data used here. Yet, ample evidence shows that the number of SNPs obeys a law of diminishing returns and a negligible difference in predictive performance has been observed between full sequence and high-density genotyping data [51, 62, 63]. Distributions of OTU abundances are always highly leptokurtic, as observed here, and statistical properties tend to depend primarily on the actual read depth rather than on the specific ecosystem [46].

Here, we have presumed that the effects on abundances are additive on the log scale. Similar models are widely used in a diversity of scenarios. For example, multiplicative models are used to accommodate fitness effects in evolutionary genetics [64] or to deal with highly leptokurtic distributions such as for abundances of microorganisms or of gene expression levels, which the log transformation addresses. In addition to the log-transformation, a widely popular choice in genetics is the threshold model [8], which assumes the presence of a continuous liability (here abundances) with an effect value of ‘0’ below a given threshold and ‘1’ otherwise. This model has the advantage of not depending on whether abundances are log-transformed or not and is also biologically sound since it is conceivable that a minimum microorganism abundance is required to trigger a particular effect. To test the robustness of the log-transformation, we simulated phenotypes such that 25% of the causative abundance observations were above the threshold (i.e., abundances were binarized) and the analysis was performed on the log transformed abundances, as before, using Bayes C. As expected, using a ‘wrong’ model for the analyses was detrimental to prediction but not dramatically (Fig. 8a). Compared to the multiplicative model, parameter estimates were affected downwards (Fig. 8b). On this basis, we conjecture that the fundamental results obtained herein should hold even if the relationship between OTU abundance and phenotype is not strictly multiplicative.

### There is room for methodological developments

Numerous studies have reported estimates of microbiability for economically important traits, e.g., [4, 16, 30, 59], but the reliability of these estimates is not known. Estimates may be affected by the estimation procedure and there are numerous alternatives to estimate $b^{2}$, including Bayes C [20], GBLUP [38], and Bayesian RKHS regression using either Bray–Curtis dissimilarities as relationship matrix [30] or with the variance–covariance from the log-transformed OTU as kinship matrix [14, 30]. Our results (Figs. 5 and 6) indicate that estimates of microbiability obtained using BayesC may be biased upwards, especially when $b^{2}$ is higher than 0.25 and the number of causative OTU is small. However, we found that estimates of $b^{2}$ derived using Bayes C were very close to zero in the null scenario (see Additional file 1: Figure S1B). Thus, models using priors from the Spike-Slab family, which consider a priori the possibility of null effects, can be used to test whether heritability or microbiability is substantial. In contrast, estimates obtained using Bayesian RKHS were slightly less biased and less sensitive to the number of causative OTU. However, estimates of variance fractions in the order ~ 0.10 were obtained even if the true variance was zero. As a result, small effects may not be distinguishable from null effects. Ramayo-Caldas [30] reported higher microbiability estimates using Bray–Curtis based kernels with Bayesian RKHS than those using the log-transformed covariance matrix of abundances. Other methods have been proposed to select variables in a context of compositional data, e.g., [65, 66]. The behavior of estimation methods for microbiability certainly merits further research.

One conclusion from this work is that it will be difficult to distinguish between some of the underlying scenarios or to identify causative OTU and SNPs, at least by using standard linear models, as was done here. The distinction between ‘Joint’ and ‘Recursive’ scenarios is of special relevance for breeding. The latter assumes partial genetic control of some causative OTU. Yet, we found that both scenarios resulted in very similar patterns in terms of predictive performance and parameter estimates (Figs. 3, 4, 5, 6). Perhaps, a more powerful approach would be to use structural equation models (SEM), which allow the inclusion of a variable both as an independent and a dependent variable. Saborio-Montero et al. [67] compared a linear bivariate (one OTU and the phenotype) model with a SEM but found few differences between models. One limitation of their approach is that one SEM was fitted for each abundance, instead of fitting several abundances simultaneously.

There is growing evidence of interactions between the microbiome and the host genome [1, 25, 68] but it is not clear which approach is optimal to statistically model this phenomenon. The interaction of the genome and the microbiome poses challenging modeling and prediction problems. A main aim of this paper was to assess how ‘standard’ linear approaches behave under these complex scenarios. Although the number of possible interactions to consider can be huge when the number of SNPs and the number of OTU is large, interactions between features in two high-dimensional sets can be modeled in a Gaussian context using co-variance functions. These functions are the Hadamard product of set-specific similarity matrices such as the Hadamard product of a SNP-derived and an OTU-derived ‘relationship’ matrix. Such an approach has already been used to model, e.g. interactions between SNPs or between SNPs and environmental covariates, e.g., [54], and here we employed this in the RKHS framework through the ${\mathbf{G}} \circ {\mathbf{B}}$ covariance matrix. Unfortunately, no improvement in prediction was attained and the model was not able to capture the covariance between genome and microbiome in the recursive scenario. None of the methods evaluated here were optimal for both inference and prediction. Thus, there is room for improvement, but this requires proper theoretical development that is beyond the scope of this work. Possibilities include extending recursive models as done, e.g., by Saborio-Montero et al. [59], or improved selection indices, as in Weishaar [69].

A wide array of penalized linear methods exists for inference and prediction, which differ in their priors [70]. We observed that Bayes C performed better than Bayesian RKHS in terms of prediction. We used a non-uniform distribution for simulating the effects of genes and OTU, specifically a gamma distribution, as is sustained by empirical and theoretical investigations, at least for gene effects [48, 50, 71]. Thus, it can be expected that the Bayes C prior fitted the simulated data better than RKHS, for which the prior was flat across SNPs and OTU. The well-known ‘no-free-lunch theorem’ [72] in computer sciences states that no method is superior in performance across all scenarios or for all tasks. In agreement with this, we found that variance component estimates from RKHS were both slightly less biased and much less sensitive to the number of causative effects (OTU or SNPs) than estimates from Bayes C. Previous work also showed that Bayes C variance components are highly sensitive to the genetic architecture of the trait [73].

### Final remarks: on using microbiome for prediction of complex traits

The utility of microbiome for the prediction of complex traits, e.g., in prospective studies or in breeding, depends crucially on its stability in time and space. Stability will likely be lower for rare OTU than for core members [13], and so the abundance of causative OTU will be relevant in this context. For instance, although measures of gastrointestinal microbiome abundances are known to be repeatable, they cannot be expected to remain stable throughout an individual’s entire life span. After weaning and under standard management conditions, e.g., constant diet and absence of antibiotic treatment, the diversity of monogastric gut microbiota increases with age of the host until its composition remains stable [34] Rumen microbial communities are highly resilient and host-specific [74, 75] but also change in early life, with the transition towards a more stable and adult-like ruminal ecosystem occurring between weaning and one year of age [76]. Therefore, for prediction purposes, we recommend the inclusion of microbial data obtained at least after weaning, preferably at adulthood. Compared to genomic data, this certainly limits the use of microbiota for prediction in breeding schemes.

## Conclusions

To conclude, this study suggests that microbiome data can significantly improve the prediction of complex phenotypes, regardless of whether some abundances are under direct genetic control or not. However, for this strategy to be successful, medium- to large-sized experiments are required and the microbiome should be relatively stable and available prior to phenotype collection. This limits the use of the microbiome for prediction in breeding schemes as compared to genome data, which can be collected at birth and remains unchanged. Nevertheless, important potential applications remain, such as predicting methane emission in cattle, obesity and feed efficiency, disease predisposition, or crop production using the soil metagenome. Overall, we show that standard linear methods can be used, in spite of the highly leptokurtic distributions observed in OTU abundances. Given the specific advantages of each of the algorithms evaluated, there is room for specific theoretical developments that combine benefits from both. Nonetheless, we argue that new models should be based on a better understanding of the relation between the microbiome and the phenotype. It seems important to quantify, even approximately, the number of taxa that affect the phenotype and to characterize the distribution of their effects, as it may affect the reliability of parameter estimates (Fig. 5). However, we are far less optimistic (e.g., Fig. 7) with regards to the identification of causative OTU, and of the putative QTN that affect relative abundances.

## Supplementary Information

**Additional file 1: Figure S1.** Comparison between flat and informative priors. Posterior distributions of heritability (black line) and microbiability (blue dashed line) in a single replicate of the ‘Joint’ scenario, $r^{2}$ = 0.25. Numbers in the panel titles are predictive accuracies for each prior and method. Overall, mildly informative priors resulted in similar predictive accuracies as for flat priors and more reasonable posterior distributions. **Figure S2.** Gibbs sampling values of heritability (black line), microbiability (red line), and correlation between genome and microbiome effects (green line) in a single replicate of the ‘Recursive’ scenario ($r^{2}$ = 0.5). The comparison is between flat and informative priors. **Figure S3.** Results with the null model using the Bayes C model. This figure shows the results with the null model using the Bayes C model when samples were permuted relative to genotypes and abundances. (A) Prediction accuracy, computed as correlation between predicted and observed phenotypes, for each of the Bayes C analyses: Cgb includes microbiome and genome; Cg includes genome data only, and Cb includes microbiome data only. (B) Estimates of heritability ($h^{2}$) and microbiability ($b^{2}$) for each of the Bayes C analyses. Data are the average of 30 replicates. **Figure S4.** Plot of the principal component analysis of the original abundance data (top left) and three simulated datasets under the ‘Recursive’ model. Each dot corresponds to a single individual and data are log-transformed. The permutation has a negligible influence on the data structure. **Figure S5.** Distributions of effects. (A) Observed and fitted (red line) distribution of abundance linear regression effects on methane emissions reported by Difford et al. [4]. (B) Comparison of gamma distributions used for sampling genetic ($\alpha$) and OTUs’ ($\omega$) effects: $\alpha \sim {\Gamma }$ ($k$ = 0.2, $\theta$ = 5) and $\omega \sim {\Gamma }$ (k = 1.4, $\theta$ = 3.8), plotted in red and black lines, respectively (Eq. 1).

## Data Availability

GitHub site https://github.com/miguelperezenciso/simubiomecontains the code, documentation and data links.
