# Correction to: Opportunities and limits of combining microbiome and genome data for complex trait prediction

**DOI:** 10.1186/s12711-021-00691-6

**Published:** 2021-12-20

**Authors:** Miguel Pérez-Enciso, Laura M. Zingaretti, Yuliaxis Ramayo-Caldas, Gustavo de los Campos

**Affiliations:** 1 ICREA, Passeig de Lluís Companys 23, 08010 Barcelona, Spain; 2 Centre for Research in Agricultural Genomics (CRAG), CSIC-IRTA-UAB-UB, 08193 Bellaterra, Barcelona Spain; 3 Animal Breeding and Genetics Program, Institute for Research and Technology in Food and Agriculture (IRTA), Torre Marimon, 08140 Caldes de Montbui, Barcelona Spain; 4 Dept. of Epidemiology & Biostatistics, and Dept. of Statistics & Probability, Michigan State University, East Lansing, MI 48824 USA

## Correction to: Genet Sel Evol (2021) 53:65 https://doi.org/10.1186/s12711-021-00658-7

Following publication of the original article [1], the authors noticed the following two errors:

1. Figures 1 and 2 are swapped, but the legends and figure numbers are correct, that is, the legend indicated for Figure 1 is in the right place but corresponds to Figure 2, and vice versa;
  and
2. In the Funding section, the statement:
  MPE is funded by Ministry of Science and Innovation-State Research Agency (AEI) Grant PID2019-108829RB-I00 is incomplete. The correct paragraph should be:
  MPE is funded by the Ministry of Science and Innovation-State Research Agency (MICIN/AEI/10.13039/501100011033) Grant PID2019-108829RB-I00.
