# Supplementary material for: Opportunities and limits of combining microbiome and genome data for complex trait prediction
Source: Genet Sel Evol. 2021 Aug 6;53:65. doi: 10.1186/s12711-021-00658-7 (PMC8344190; doi:10.1186/s12711-021-00658-7)

***Fig S1: Comparison between flat and informative priors****. Posterior distributions of heritability (black line) and microbiability (blue dashed line) in a single replicate of the Joint scenario, r2 = 0.25. Numbers in panel titles are predictive accuracies for each prior and method. Overall, mildly informative priors resulted in similar predictive accuracies as for flat priors and more reasonable posterior distributions.*

***
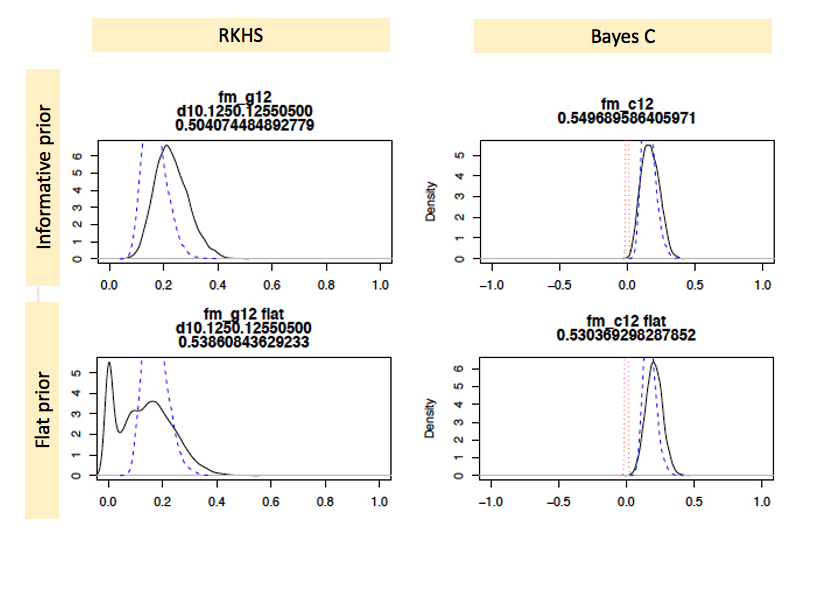
***

***Fig S2: Gibbs sampling values*** *of heritability (black line), microbiability (red line), and correlation between genome and microbiome effects (green line) in a single replicate of the Recursive scenario (r^2^ = 0.5).*

***
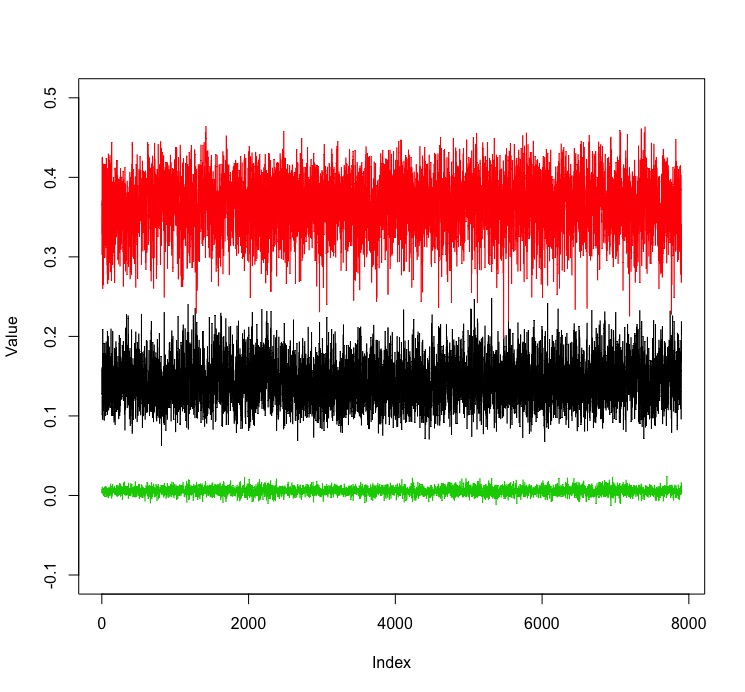
***

***Fig S3: Results with the null model using the Bayes C model****, i.e., when samples were permuted relative to genotypes and abundances.* ***A)*** *Prediction accuracy, computed as correlation between predicted and observed phenotypes, for each of the Bayes C analyses: Cgb includes microbiome and genome; Cg includes genome data only, and Cb includes microbiome data only;* ***B)*** *Estimates of heritability (h^2^) and microbiability (b^2^) for each of the Bayes C analyses. Data are average of 30 replicates.*


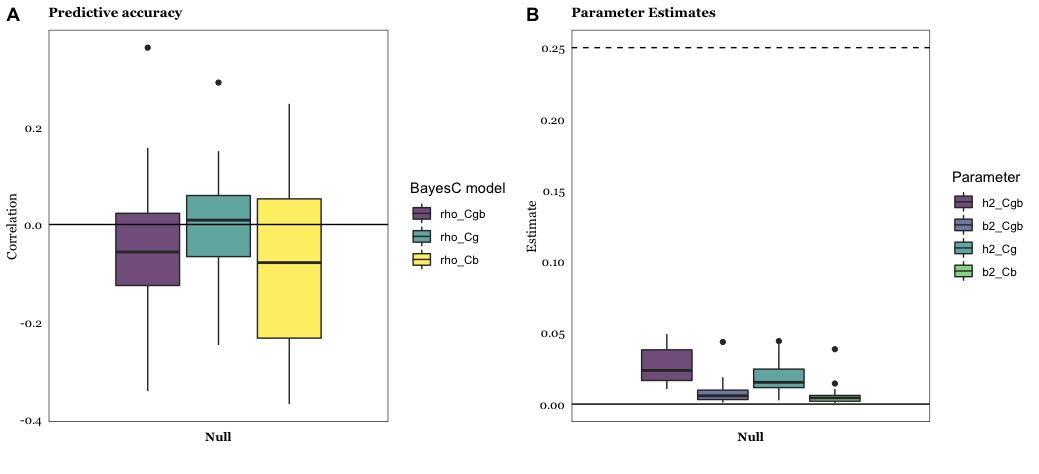


***Fig S4:*** ***Principal Component Analysis plot of the original abundance data*** *(top left) and three simulated datasets under the Recursive model. Each dot corresponds to a single individual, data are log-transformed. The permutation induced has a negligible influence on the data structure.*


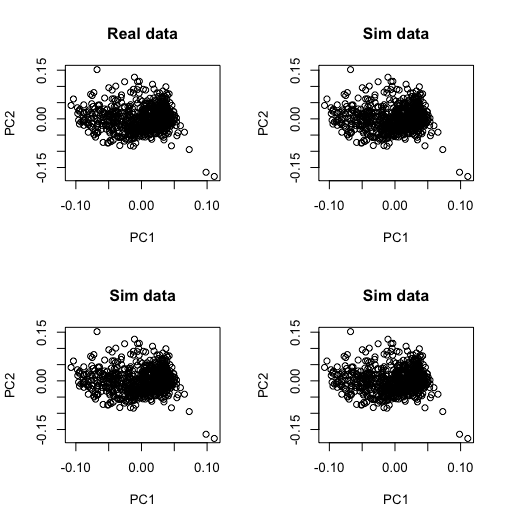


***Fig S5: Distributions of effects. A)*** *Observed and fitted (red line) distribution of abundance linear regression effects on methane emissions reported by Difford et al. [4].* ***B)*** *Comparison of gamma distributions used for sampling genetic (α) and OTUs’ (ω) effects: α ~ Γ(k=0.2, θ=5) and ω ~ Γ(k=1.4, θ=3.8), plotted in red and black lines, respectively (Eqn. 1).*

**A B**


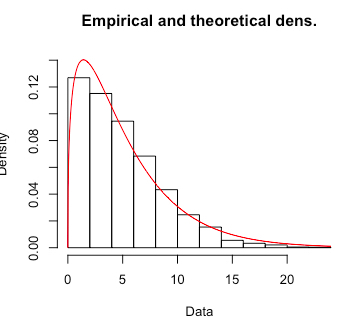

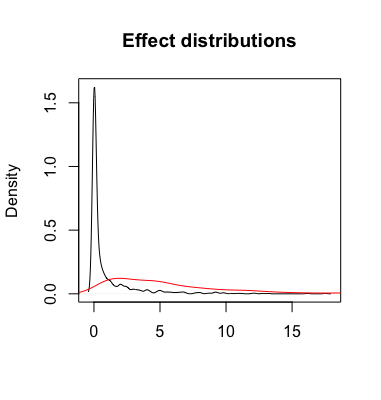

Supplement: Supplementary file 1 — Additional file 1: Figure S1. Comparison between flat and informative priors. Posterior distributions of heritability (black line) and microbiability (blue dashed line) in a single replicate of the ‘Joint’ scenario, $r^{2}$r2 = 0.25. Numbers in the panel titles are predictive accuracies for each prior and method. Overall, mildly informative priors resulted in similar predictive accuracies as for flat priors and more reasonable posterior distributions. Figure S2. Gibbs sampling values of heritability (black line), microbiability (red line), and correlation between genome and microbiome effects (green line) in a single replicate of the ‘Recursive’ scenario ($r^{2}$r2 = 0.5). The comparison is between flat and informative priors. Figure S3. Results with the null model using the Bayes C model. This figure shows the results with the null model using the Bayes C model when samples were permuted relative to genotypes and abundances. (A) Prediction accuracy, computed as correlation between predicted and observed phenotypes, for each of the Bayes C analyses: Cgb includes microbiome and genome; Cg includes genome data only, and Cb includes microbiome data only. (B) Estimates of heritability ($h^{2}$h2) and microbiability ( [file 12711_2021_658_MOESM1_ESM.docx]
